# Antigen-Specific Regulatory T Cell Therapy in Autoimmune Diseases and Transplantation

**DOI:** 10.3389/fimmu.2021.661875

**Published:** 2021-05-14

**Authors:** Claudia Selck, Margarita Dominguez-Villar

**Affiliations:** Faculty of Medicine, Imperial College London, London, United Kingdom

**Keywords:** regulatory T cells, Tregs, antigen-specific Tregs, autoimmune disease (AD), therapy, transplantation

## Abstract

Regulatory T (Treg) cells are a heterogenous population of immunosuppressive T cells whose therapeutic potential for the treatment of autoimmune diseases and graft rejection is currently being explored. While clinical trial results thus far support the safety and efficacy of adoptive therapies using polyclonal Treg cells, some studies suggest that antigen-specific Treg cells are more potent in regulating and improving immune tolerance in a disease-specific manner. Hence, several approaches to generate and/or expand antigen-specific Treg cells *in vitro* or *in vivo* are currently under investigation. However, antigen-specific Treg cell therapies face additional challenges that require further consideration, including the identification of disease-relevant antigens as well as the *in vivo* stability and migratory behavior of Treg cells following transfer. In this review, we discuss these approaches and the potential limitations and describe prospective strategies to enhance the efficacy of antigen-specific Treg cell treatments in autoimmunity and transplantation.

## Introduction

Treg cells play an essential role in the maintenance of immune homeostasis by inhibiting pathological responses towards self-antigens and controlling potentially harmful inflammatory reactions following infections. While different T cell populations with immunosuppressive capacity have been described in recent years including type 1 regulatory T (Tr1) cells and T helper 3 (Th3) cells (1, 2), CD4^+^CD127^-^CD25^high^ T cells that express the transcription factor forkhead box P3 (FOXP3) remain the most studied Treg subset to date and thus, will be the main focus of this review. In healthy individuals, FOXP3^+^ Treg cells are generated both in the thymus (tTreg) upon intermediate avidity interaction of developing thymocytes with self-peptides (3) and in the periphery (pTreg) during antigen encounter of conventional naïve CD4^+^ T cells in tolerogenic environments, such as the presence of transforming growth factor beta (TGF-ß) and interleukin-2 (IL-2) (4, 5). Although specific biomarkers that allow the distinction between tTreg and pTreg cells are currently not available, it is assumed that the antigen specificities of these Treg subsets differ substantially due to their distinct developmental origin (6, 7). The T cell receptor (TCR) repertoire of tTreg cells is skewed toward autoantigen recognition and hence, they predominantly maintain self-tolerance by preventing immune responses against the body’s own tissues and organs (8). In contrast, pTreg cells mainly recognize non-self-antigens derived from commensal bacteria, infectious pathogens or ingested food and thus, sustain mucosal tolerance, inhibit inflammation-induced tissue damage and avert allergic reactions (5, 9–11). Importantly, various critical questions about the maintenance and function of these antigen-specific Treg cells remain unanswered, involving their *in vivo* cellular targets, the molecular pathways triggering their activation and the underlying mechanisms controlling their suppressive function.

Considering the crucial functions of FOXP3^+^ Treg cells in maintaining a healthy state, it is not surprising that defects in their biology can lead to detrimental disruptions of immune homeostasis. In particular, multiple preclinical and human studies have demonstrated that a number of Treg-specific defects are associated with the development of several autoimmune disorders (AID) such as type 1 diabetes (T1D) (12–14), rheumatoid arthritis (RA) (15, 16), multiple sclerosis (MS) (17–19), systemic lupus erythematosus (SLE) (20) and psoriasis (21, 22). These Treg-specific defects include reduced proliferative and migratory capabilities (21, 23) as well as lower expression levels of essential Treg markers, including FOXP3 and CD25 (24–27). Moreover, Treg cells isolated from patients with several AID exhibit impaired immunosuppressive functions associated with reduced expression of anti-inflammatory molecules such as IL-10, cytotoxic T lymphocyte antigen 4 (CTLA-4), T cell immunoglobulin and mucin domain-containing 3 (Tim-3) and indoleamine 2,3-dioxygenase (IDO) (16, 28–30), and increased production of pro-inflammatory cytokines such as interferon gamma (IFN-γ) and IL-17 (13, 19). Some studies indicate that these deficiencies are predominantly observed in the naïve Treg compartment which is presumed to be largely comprised of tTreg cells (18, 31). Nonetheless, it is still unclear whether defects of Treg cell numbers and/or function in human AID are limited to disease-associated antigen-specific Treg cells or affect polyclonal Treg populations since the antigen specificity of impaired Treg cells remains insufficiently characterized.

## Polyclonal vs. Antigen-Specific Treg Therapies

**Figure 1:**
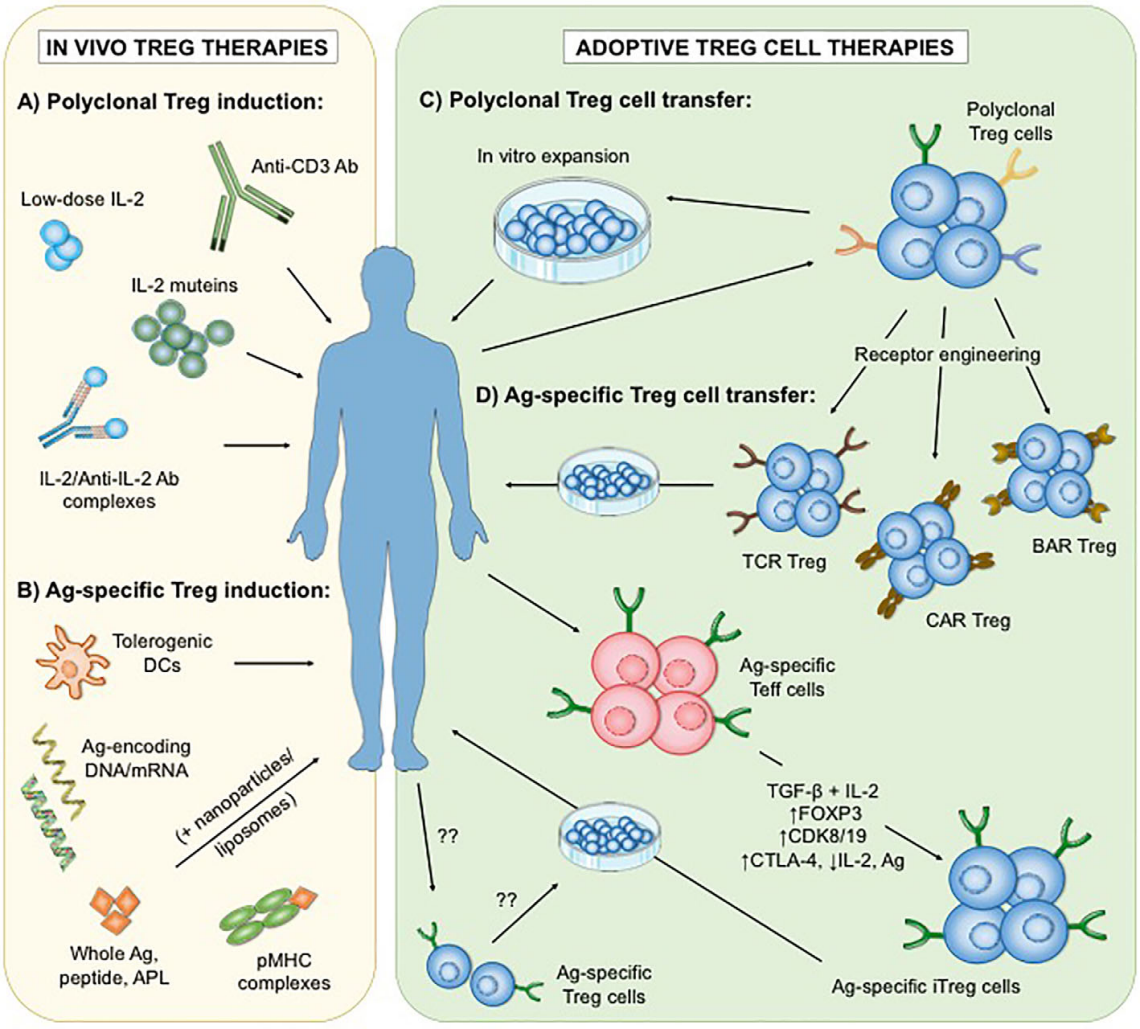
Different approaches of polyclonal and antigen-specific Treg cell-based therapies. To date, two main strategies have been developed: the administration of immunomodulatory agents that enhance the number and/or function of Treg cells *in vivo* **(A, B)**, and the adoptive transfer of *in vitro* expanded Treg cells **(C, D)**. Interventions that increase polyclonal endogenous Treg cells *in vivo* involve low-dose interleukin-2 (IL-2), mutant IL-2, IL2/Anti-IL-2 Ab complexes as well as selective depletion of Teff cells by Anti-CD3 Ab **(A)**. In contrast, applications of antigen-based treatments could lead to the enhancement of antigen-specific Treg subsets **(B)**. On the other hand, adoptive Treg cell therapies rely on the optimal isolation and expansion of Treg cells *in vitro*. Thus far, clinical trials in autoimmunity have only utilized expanded polyclonal Treg cell populations **(C)**. However, antigen-specific Treg cells can be generated *in vitro* **(D)** by genetic insertion of synthetic receptors (including engineered T cell receptors (TCR), chimeric antigen receptors (CAR) or B cell antibody receptors (BAR)), or by transformation of antigen-specific effector T (Teff) cells into induced Treg (iTreg) cells *via* stimulation in the presence of transforming growth factor beta (TGF-ß) and IL-2, transgenic FOXP3 overexpression, blockade of cyclin-dependent kinase 8 (CDK8) and CDK19 signaling, or a combination of cytotoxic T lymphocyte antigen 4 (CTLA-4) overexpression, IL-2 ablation and antigenic stimulation. The isolation and expansion of endogenous antigen-specific Treg cells remains technically challenging. Ag, antigen; DCs, dendritic cells; APL, altered peptide ligands; pMHC, peptide-major histocompatibility complex.

While new key factors and mechanisms underlying Treg biology continue being elucidated, Treg cell-based therapies have been proposed to be a promising strategy for the re-establishment of immune tolerance in individuals with AID, allergies or organ transplantation (32–35). These treatments currently involve either the adoptive transfer of *in vitro* expanded Treg cells, or the administration of immunomodulatory interventions that promote the expansion and/or function of Treg cells *in vivo* (**Figure 1**). Notably, both of these applications have the potential to promote Treg-mediated immune regulation in a polyclonal or antigen-specific manner with each harboring their own advantages and limitations.

The success of adoptive Treg cell therapies depends on multiple critical factors, including the optimal source of Treg cells, appropriate cell isolation and expansion procedures as well as optimal cell dose and number of infusions administered. First early-phase clinical trials investigating the safety of autologous or allogeneic Treg transfer demonstrated good toxicity profiles in patients with T1D (32, 36–38), MS (39), Crohn’s disease (33), graft versus host disease (GvHD) (40–42) and kidney/liver transplantation (34, 43–46). In addition, some of these interventions induced signs of disease improvement which supported the investigation of treatment efficacy in larger trials (36–38, 41, 46). Importantly, while these initial human studies adopted somewhat comparable cell enrichment and culture protocols, all of them utilized polyclonal Treg subsets that exhibit a plethora of different TCR specificities. The potential therapeutic benefit of polyclonal Treg population infusion relies on bystander immunosuppression which allows regulation by activated Treg cells through antigen-independent processes (47). Since polyclonal Treg cells undergo extensive activation and expansion *in vitro* prior to adoptive transfer, it is possible that they are capable of implementing this bystander effect. Therefore, a number of ongoing clinical studies are using polyclonal Treg cells for the treatment of AID including T1D (NCT02772679, NCT03444064), ulcerative colitis (NCT04691232) and Pemphigus (NCT03239470). However, growing evidence from animal models indicates that antigen-specific Treg cells may be more efficient in controlling pathological immune responses in a disease-specific manner (**Table 1**) (48–64). This is likely due to the migration of infused Treg cells towards tissues of cognate antigen exposure (49, 63) leading to more potent and localized control of inflammation with reduced risks of broad immunosuppression and associated adverse events. Moreover, the enhanced trafficking of antigen-specific Treg cells to target tissues presumably allows the administration of lower Treg cell numbers than polyclonal approaches, potentially facilitating the obtention of these cell numbers in standard *in vitro* expansion protocols. Nevertheless, the purification and expansion of disease-relevant antigen-specific Treg cells remains technically challenging because of their very low frequency in the peripheral blood (65). Therefore, current efforts are focusing on the generation of antigen-specific Treg cells *in vitro* by transformation of antigen-specific effector T (Teff) cells into cells with suppressive capacity (66–68), or genetic insertion of synthetic antigen receptors with disease-relevant antigenic specificities into isolated Treg populations (53, 64, 69) (**Figure 1D**).

**Table 1:** Pre-clinical studies demonstrating increased efficacy of antigen-specific adoptive Treg cell therapies for AID and transplantation.

Disease	Model	Antigen-specific Treg population	Evidence of superior function	Ref.
T1D	(BDC2.5) NOD mice	CD4^+^ CD25^+^ T cells from TCR-transgenic BDC2.5 mice expanded *in vitro* with BDC peptide and NOD DCs	Efficient inhibition of diabetogenic T cell-induced diabetes in NOD mice (no suppression with polyclonal CD4^+^ CD25^+^ NOD Treg cells)	(48)
T1D	(BDC2.5) NOD mice	CD4^+^ CD25^+^ T cells from TCR-transgenic BDC2.5 mice expanded *in vitro* with anti-CD3/CD28 beads	Enhanced suppression + reversal of diabetogenic T cell-induced diabetes in NOD.RAG-/- or NOD CD28-/- mice (only slight delay of disease with 4-fold higher numbers of polyclonal CD4^+^ CD25^+^ NOD Treg cells)	(49)
RA	DBA1 mice	CD4^+^ T cells transduced with FOXP3 and a TCR of a CIA-associated T cell clone	Effective inhibition + reversal of CIA (no effect with FOXP3-transduced CD4^+^ T cells without antigen specificity)	(50)
MS	(Tg4) B10.PL mice	CD4^+^ CD25^+^ T cells from TCR-transgenic Tg4 mice expanded *in vitro* with anti-CD3/CD28 beads	Potent inhibition + amelioration of MBP- or PLP-induced EAE (no effect with polyclonal B10.PL Treg cells)	(51)
MS	(2D2) C57Bl/6 mice	HDR-edited FOXP3-overexpressing T cells (edTreg) from TCR-transgenic 2D2 mice	Better suppression of Teff proliferation *in vivo* in MOG-induced EAE compared to polyclonal C57Bl/6 edTreg cells	(52)
MS	C57Bl/6 mice	MOG-specific CAR-engineered CD4^+^ T cells with transgenic FOXP3 expression	Increased migration into the brain + better control of MOG-induced EAE than MOCK-treated FOXP3^+^ T cells	(53)
Autoimmune Neuropathy	Lewis rats	CD4^+^ CD25^+^ T cells from rats expanded *in vitro* with PNM and IL-2	Amelioration of PNM-induced EAN (no effects with CD4^+^ CD25^+^ T cells expanded with irrelevant autoantigen)	(54)
Colitis	TNP-Tg BALB/c mice	CAR-engineered CD4^+^ CD25^+^ Treg cells specific for TNP	Protection from TNBS-induced colitis (no effect with control CAR Treg cells)	(55)
Colitis	CEABAC mice	CAR-engineered CD4^+^ CD25^+^ Treg cells specific for CEA	Enhanced colon homing + more efficient amelioration of Teff-mediated and AOM-DSS-induced colitis compared to control CAR Treg cells	(56)
AIG	(TxA23) BALB/c mice	TGF-ß-induced iTreg cells generated from CD4^+^ T cells of TxA23 mice	Prevention of Teff cell-induced AIG (no suppression with polyclonal BALB/c iTreg cells)	(57)
Skin transplantation	BRG mice	CAR-engineered human CD4^+^ CD25^+^ Treg cells specific for HLA-A2	Reduced graft injury in a human skin xenograft model compared to polyclonal Treg cells	(58)
Skin transplantation	NRG mice	CAR-engineered human CD4^+^ CD25^+^ Treg cells specific for HLA-A2	Superior inhibition of allospecific immune responses than polyclonal Treg cells in human skin xenograft model	(59)
Skin transplantation/GvHD	NSG mice	CAR-engineered human CD8^+^ CD45RC^low^ Treg cells specific for HLA-A2	More potent suppression of immune responses than control CAR Treg cells	(60)
GvHD	(OVA Tg) C57Bl/6 mice	TGF-ß-induced OVA-specific iTreg cells generated from CD4^+^ T cells of OT-II mice	Better prevention of GvHD than polyclonal iTreg cells	(61)

T1D, type 1 diabetes; NOD, non-obese diabetic; RA, rheumatoid arthritis; MS, multiple sclerosis; AIG, autoimmune gastritis; TCR, T cell receptor; DCs, dendritic cells; CIA, collagen-induced arthritis; HDR, homology-directed repair; MOG, myelin oligodendrocyte glycoprotein; MBP, myelin basic protein; PLP, proteolipid protein; EAE, experimental autoimmune encephalomyelitis; CAR, chimeric antigen receptor; PNM, peripheral nerve myelin; EAN, experimental autoimmune neuritis; TNP, 2,4,6-trinitrophenol; TNBS, 2,4,6-trinitrobenzene sulphonic acid; CEA, carcinoembryonic antigen; AOM-DSS, azoxymethane-dextran sodium sulfate; TGF-ß, transforming growth factor beta; HLA, human leukocyte antigen; GvHD, graft versus host disease; OVA, ovalbumin.

## Generation of Antigen-Specific Treg Cells by Antigen-Specific Effector T Cell Engineering

Similar to the development of pTreg cells *in vivo* several studies have demonstrated that both murine and human Treg cells can be generated from naïve CD4^+^ T cells *in vitro* when they are stimulated in the presence of TGF-ß and IL-2 (induced Treg, iTreg) (66, 70). Hence, isolated antigen-specific effector T cells (Teff) could serve as a useful source to generate antigen-specific iTreg cells for adoptive cell therapy. However, it has become clear that the phenotype and function of iTreg cells is not properly maintained under inflammatory conditions (71–73). This is clinically relevant as iTreg cells might be able to regain their pro-inflammatory characteristics *in vivo* and contribute to an augmented autoimmune response especially considering the inflammatory environment where they will be re-infused in. Thus, other strategies to re-program Teff lymphocytes into Treg cells have been developed including transgenic overexpression of FOXP3 *via* lentivirus-based techniques (74–78). While several studies demonstrated that FOXP3-transduced Teff cells exhibit Treg-like phenotypes and immunosuppressive functions, the random insertion of FOXP3 at different lentiviral integration sites might entail potential safety risks due to the heterogeneity of the final clinical product. Therefore, more advanced genetic tools, such as CRISPR/Cas9 or TALEN, have been recently utilized to generate FOXP3-expressing Teff cells *via* homology-directed repair-based gene editing (52, 79). Moreover, a CRISPR-based system has been shown to successfully repair the FOXP3 gene in T cells from IPEX (immune dysregulation polyendocrinopathy enteropathy and X-linked) syndrome patients (79). In addition, recent data have demonstrated the feasibility of generating human antigen-specific Treg cells from tetramer-enriched Teff populations by introduction of a transgenic FOXP3 promoter *via* TALEN and adeno-associated virus-based editing (52). It is noteworthy that Teff cells can also acquire Treg-like characteristics by FOXP3-independent approaches, including blockade of cyclin-dependent kinase 8 (CDK8) and CDK19 signaling pathways (67), as well as by a combination of CTLA-4 overexpression, IL-2 ablation and antigenic stimulation (80). All of these strategies were able to confer immunosuppressive functions to both naïve and activated Teff cells which retained their anti-inflammatory properties *in vivo* when transferred into different mouse models of autoimmunity (52, 67, 80). Nonetheless, it remains to be determined whether these applications would have clinical utility in human AID.

## Generation of Antigen-Specific Treg Cells by Genetic Engineering

A different approach to generate antigen-specific Treg cells *in vitro* involves the alteration of polyclonal Treg specificities by genetic introduction of synthetic receptors, including engineered TCRs and chimeric antigen receptors (CARs). For example, Treg cells transduced with an exogenous TCR isolated from human islet-specific CD4^+^ T cell clones possess more potent antigen-specific suppressive capacities than polyclonal Treg populations (64). Furthermore, adoptive transfer of Treg cells engineered with a TCR specific for myelin basic protein can efficiently improve experimental autoimmune encephalitis (EAE) (81). Different reports from animal models of T1D and RA also demonstrate that TCR engineering can be successfully combined with the transduction of FOXP3 in order to convert Teff lymphocytes into immunosuppressive antigen-specific Treg-like cells (68, 82). Although these preclinical studies are encouraging, the translation of TCR-engineered Tregs into the clinic is somewhat limited by their major histocompatibility complex (MHC) restriction and the need to isolate and identify antigen-specific and disease-relevant TCRs.

On the other hand, the development of chimeric antigen receptors (CARs) enables the generation of engineered Treg cells that recognize their antigen directly (including whole proteins) in a non-MHC restricted manner (83). CARs consist of an extracellular single-chain variable antibody fragment (scFv) fused with an intracellular CD3 activation domain and (potentially multiple) co-stimulation domains. While it has been suggested that integration of the co-stimulatory molecule CD28 is essential for potent CAR Treg functions (84), the optimal design of CAR Treg cells is still under ongoing investigation (85). Nevertheless, based on their successful application and tolerable safety profiles in cancer treatments (86), the transduction of CARs may be considered a promising approach for future clinical administration of antigen-specific Treg cells in AID and transplantation. Notably, whereas CARs possess a higher affinity for their cognate antigen than TCRs, data suggest that CARs require a greater density of antigen for their activation (87, 88) [reviewed in (89)]. Thus, the use of CAR Treg cells might be more beneficial for clinical settings where the relevant antigen is highly expressed in the target site while TCR-engineered Treg cells are potentially more efficacious in diseases associated with low antigen expression levels.

Initial studies in autoimmunity reported that CAR-engineered Treg cells specific for 2,4,6-trinitrophenol (TNP) can efficiently reduce murine colitis whereas this was not observed with irrelevant CAR Treg cells (55, 90). Similar results were obtained in two different experimental colitis models that utilized CAR Treg cells recognizing carcinoembryonic antigen (CEA) and confirmed the superior immunosuppressive function of CEA-specific Treg cells compared to non-specific control Treg cells. Moreover, histological analysis revealed that only CEA-CAR Treg cells were able to migrate to the inflamed colon of diseased animals (56). Furthermore, myelin oligodendrocyte glycoprotein (MOG)-specific CAR Treg cells have been shown to better control EAE than sham-treated Treg cells. In this study, CAR engineering was combined with the transgenic expression of FOXP3 in CD4^+^ Teff cells resulting in MOG-specific immunosuppressive Treg cells that were able to home to the brain, to decrease EAE symptoms and to mediate protection from a second EAE challenge using pertussis toxin and complete Freund’s adjuvant (53). In addition, HLA-A2 CAR-expressing (CD4^+^ or CD8^+^) Treg cells have been used in different pre-clinical studies of skin transplantation demonstrating superior suppression of human skin graft rejection and reduced GvHD in humanized mouse models (58–60). A phase 1/2a trial is currently examining the safety of HLA-A2 CAR-engineered autologous Treg cells in kidney transplantation (NCT04817774).

Strategies utilizing genetically engineered Treg cells with B cell antibody receptors (BARs) are also under ongoing investigation. Instead of the extracellular scFv used in CARs, BARs contain an antigen or antigen fragment that can be recognized by B cell receptors (BCRs) on inflammatory antibody producing B cells (91). Like CAR Treg cells, BAR Tregs are not MHC restricted and initial results demonstrated potent immunosuppression in mouse models of allergy (92) and hemophilia (69). Finally, although only currently studied in the context of T cells, and not Tregs, chimeric autoantibody receptor (CAAR) engineering could provide an additional approach to directly target autoreactive B cells in AID (93).

## *In Vivo* Treg Cell-Based Therapies

While the development and conduction of adoptive Treg cell therapies are costly and laborious, immunomodulatory drugs that target key molecules of Treg maintenance have the potential to improve Treg-mediated immune tolerance *in vivo*. These treatments can increase the expansion and/or function of polyclonal or antigen-specific Treg subsets depending on the underlying mechanism of action targeted (**Figures 1A, B**, respectively). Due to the higher expression of CD25 (the alpha chain of IL-2 receptor) on Treg cells compared to Teff cell populations, interventions that promote Treg-specific IL-2 signaling constitute an attractive approach to improve the performance of the whole endogenous Treg cell pool. Nonetheless, because of its wide range of cellular targets including CD4^+^ and CD8^+^ effector T cells and natural killer cells, different strategies that avoid bystander activation of these pro-inflammatory subsets have been developed. These include the treatment with low-dose IL-2 (94–96), engineered IL-2 muteins (97–99) and IL-2/IL-2 antibody complexes (100, 101) that predominantly bind to CD25 over CD122 (IL-2R beta chain) and hence, preferentially induce the expansion of Treg population over Teff lymphocytes. Besides IL-2, some studies suggest that multiple other cytokines can promote the induction and/or suppressive function of antigen-specific Treg cells, including IL-4 (102), IL-5 (103), IL-7 (104), IL-12 (105), IL-15 (106) and IFN-γ (107). Furthermore, Treg cell homeostasis relies on several other signaling molecules that can be targeted to increase Treg cell performance *in vivo*, like mammalian target of rapamycin (mTOR) (108), phosphatase and tensin homolog (PTEN) and protein phosphatase 2A (PP2A) (109–111) as well as essential metabolites (e.g. kynurenine and adenosine) (112, 113). The activation of costimulatory [such as tumor necrosis factor receptor 2, TNFR2 (114)] or co-inhibitory receptors including T cell immunoreceptor with Ig and ITIM domains (TIGIT) (115) or programmed cell death 1 (PD-1) (116), predominantly expressed on the surface of Treg cells, also have the potential to promote the expansion and/or function of certain Treg subsets more selectively (117). In addition, interventions that preferentially inhibit pathogenic T cells over Treg cells could be beneficial for the amelioration of autoimmunity or graft rejection. For example, anti-CD3 antibody-mediated improvement of immune tolerance in animal models and patients with AID has been associated with the promotion of Treg cells, partially by selective Teff cell depletion (118, 119).

In contrast to these non-specific immunomodulatory therapies, multiple studies suggest that treatment with disease-associated antigens can lead to the induction of antigen-specific Treg cells without the risk of broad immunosuppression (120–123). Several promising strategies to administer different kinds of antigenic drugs have been shown to inhibit inflammation and disease in preclinical models, but antigen delivery in human studies did not result in the same level of clinical improvements to date, with some of them even leading to worsening of disease (124) (reviewed in (125)). However, some reports detected signs of therapeutic benefit and induced immunotolerance which was associated with the expansion of FOXP3^+^ Treg cells (122, 123). In the phase 1/2 Pre-Point trial islet autoantibody-negative children that were genetically at risk to develop T1D received oral insulin for 3-18 months. Interestingly, insulin treatment led to an immune response without unwanted hypoglycemia and induced insulin- and proinsulin-responsive T cells that exhibited characteristics of Treg cells, including FOXP3 expression and lack of CD127 and pro-inflammatory cytokines (122). Another study reported improved C-peptide retention and lower insulin use in new-onset T1D patients that were intradermally injected with an immunodominant proinsulin peptide compared to a placebo group (123). This clinical benefit was associated with increased FOXP3 expression in Treg cells and higher levels of IL-10 secretion following proinsulin stimulation. It is worth mentioning that antigen administration in animal models of T1D, EAE and collagen-induced arthritis have also resulted in the generation of immunosuppressive IL-10 producing Tr1-like cells (120, 126) which might as well be beneficial in human AID (127, 128).

## Challenges and Improvements of Antigen-Specific Treg Therapies

In order to develop efficient antigen-specific Treg cell-based treatments, disease-associated autoantigens must be well identified and characterized. However, this has not been achieved for many AID, including MS and psoriasis. The choice of the most appropriate antigen is also limited by possible antigen spreading following initial tissue damage, which could hinder the success of therapies that are based on a single antigen. This hurdle could potentially be overcome by targeting multiple self-antigens at the same time (if applicable). Nevertheless, some studies suggest that the exact definition of disease-initiating antigens might not always be necessary as long as the administered intervention leads to the accumulation of activated Treg cells in the affected inflammatory tissues that can induce other immunoregulatory populations in a contact-independent manner. This ‘infectious tolerance’ has been observed in a murine model of colitis where TNP-specific CAR Treg cells were able to reduce 2,4,6-trinitrobenzene sulphonic acid (TNBS)-induced colitis (55). Localized bystander suppression could be further supported by the transgenic introduction of appropriate surface molecules that are necessary for the migration of activated Treg cells into disease-specific inflamed sites. While critical signals of Treg cell trafficking to specific tissues remain insufficiently described, previous studies suggest that lymphocytes require the expression of the chemokine receptor CXCR3 in order to home to the brain of MS patients and the pancreatic islets of patients with T1D (129, 130). Thus, both efficacy and tolerability of Treg cell administration in these AID could be enhanced by engineering CXCR3^+^ tissue-specific Treg cells. On the other hand, patients suffering from psoriasis might benefit from Treg cells expressing the homing receptors CCR4 and cutaneous lymphocyte antigen (CLA) which are necessary for migration into the skin (131, 132).

Importantly, uncertainties about the safety of Treg cell infusions and *in vivo* immunomodulatory interventions still remain and have to be investigated with caution. In particular, the *in vivo* maintenance and suppressive function of *in vitro* generated (polyclonal or antigen-specific) Treg populations is an essential factor for the toxicity and efficacy of adoptive cell therapies. While small molecules have been shown to enhance the stability of iTreg cells *in vitro*, gene editing tools could be utilized to generate Treg cell populations with better resistance to pathological Treg plasticity in inflammatory environments (133). Such potential strategies could include overexpression of FOXP3 as aforementioned, or the knockout of molecules involved in pro-inflammatory signaling pathways present in inflamed tissues of autoimmunity. Notably, although the underlying mechanisms of Treg deficiencies in many AID are not well understood, human studies have reported that cytokines like IL-12 and IL-6 can induce defective Treg functions *in vitro* (19, 134). Hence, ablation of receptors that bind these cytokines might avoid pathological Treg instability following adoptive transfer. Moreover, genetic engineering approaches could be utilized to integrate suicide gene cassettes that can be activated in the case of disease augmentation or severe adverse events caused by harmful immune suppression, such as cancer development or chronic infections (135).

On the other hand, combination therapies of Treg cell transfer with immunomodulatory drugs that reduce autoimmune inflammation or support Treg maintenance could reduce the risk of Treg instability *in vivo*. Recently, a report demonstrated that combinatory intervention with anti-CD3 antibody enabled improved engraftment of autoantigen-specific Treg cells in the islets of a mouse model of T1D (136). The potential of anti-CD3 combination has been further confirmed in the context of antigenic peptide-based therapies with increased expansion of FOXP3^+^ insulin-specific Treg cells and more potent remission of murine autoimmune diabetes upon nasal administration of proinsulin combined with anti-CD3 treatment (137). In order to minimize the risks of severe side effects caused by immunomodulatory drugs, combination strategies that support the *in vivo* maintenance of transferred Treg cells more selectively can also be envisioned. For example, engineering of antigen-specific Treg cells with a mutant IL-2 receptor might enable specific potentiation of these infused cells in response to mutant IL-2 administration and thereby, avoid the activation of pro-inflammatory cells by wild type IL-2 (138).

A major obstacle for the development of successful antigen-specific Treg therapies is the substantial level of Treg cell heterogeneity demonstrated by the expression of different lineage-defining transcription factors, such as T-box expressed in T cells (T-bet) (139), GATA-3 (140) or retinoic acid receptor-related orphan receptor gamma (RORγt) (141), and varying levels of cell surface molecules, including co-inhibitory/co-stimulatory receptors such as PD-1 (142) and inducible T cell costimulator [(ICOS) (143)], as well as chemokine receptors including CXCR3 and L-selectin [(CD62L) (144–146)]. In addition, Treg cells can mediate their immunosuppressive effects *via* numerous mechanisms involving the secretion of anti-inflammatory cytokines (147–150), IDO (151) and granzymes (152, 153), the actions of the ectoenzymes such as CD39 and CD73 (154) and multiple inhibitory molecules, such as PD-1 (155) and CTLA-4 (156, 157). This suggests that at a given time point distinct subpopulations of FOXP3^+^ Treg cells can be identified in an individual with specialized functions and maintenance requirements which might depend on their developmental origin, the type of immune response they are controlling (Th1, Th2, or Th17-mediated inflammation) (144), or the tissue they reside in. Tissue-resident Treg cells have been found in multiple non-lymphoid tissues and organs of healthy individuals (e.g. the skin, gut, lungs, liver, adipose tissue and skeletal muscle) where they can control local inflammation, but also contribute to normal tissue homeostasis during non-inflammatory settings *via* mechanisms that are independent of their immunosuppressive functions (158–160). However, the critical maintenance factors and characteristics of tissue-resident Treg cells during health and autoimmunity are still largely unknown. Hence, it is uncertain whether antigen-specific iTreg cells or antigen-based treatments can induce tissue-specific mechanisms of Treg-mediated immune regulation and tissue homeostasis.

Moreover, the underlying causes of numerical and/or functional deficiencies of antigen-specific Treg cells in AID are not well understood and might differ between patients suffering from similar disease symptoms. This is a particularly important factor in the context of autologous adoptive Treg cell therapy as the administration of potentially defective Treg cells might not result in a desired therapeutic outcome. Hence, it is crucial to identify specific Treg defects in an individual and repair affected pathways during the *in vitro* generation/expansion of antigen-specific Treg cells before adoptive transfer. This personalized strategy could include genetic editing of molecules involved in Treg survival and fitness (such as pathways involved in IL-2 signaling and FOXP3 expression) as well as the insertion of potentially underexpressed chemokine receptors (e.g. CXCR3, CCR4, CLA) in order to increase their capacity to migrate into disease-relevant tissues.

## Conclusions

In order to develop safe and efficacious antigen-specific Treg therapies, further in-depth studies of the biology of human Treg cells during physiological homeostasis and autoimmune pathogenesis are needed. This requires new strategies to characterize distinct Treg subsets, better approaches to identify disease-relevant antigens and Treg defects as well as optimized tools to investigate clinical outcomes. In particular, new Treg biomarkers and technologies which can monitor the migratory behavior and function of infused or endogenous Treg cells *in vivo* are necessary to identify potential pitfalls that might limit therapeutic benefits. Moreover, it is conceivable that patients with AID might require subject-specific Treg-based treatments that rely on the identification of the individual’s underlying Treg deficiency. Nonetheless, the limitations of autologous Treg cell therapies could be circumvented by the use of allogeneic Treg populations with optimal MHC matching. In addition, the creation of universal Treg donor lines by genetic alterations of MHC molecules constitutes a possible strategy that deserves further investigation (discussed in (161)). Together with the ongoing efforts to develop technologies to optimally engineer human Tregs, future studies on the molecular and cellular mechanisms that control human Treg function, stability and maintenance will be critical to optimize current Treg cell-based treatments and to identify new Treg-specific targets amenable to therapeutic intervention.

## Author Contributions

CS and MD-V wrote the manuscript. All authors contributed to the article and approved the submitted version.

## Conflict of Interest

The authors declare that the research was conducted in the absence of any commercial or financial relationships that could be construed as a potential conflict of interest.
